# Knowledge and skills used for clinical decision-making on childbirth interventions: A qualitative study among midwives in the Netherlands

**DOI:** 10.18332/ejm/151653

**Published:** 2022-09-01

**Authors:** Dirkje C. Zondag, Tamar M. van Haaren-Ten Haken, Pien M. Offerhaus, Veronique Y. F. Maas, Marianne J. Nieuwenhuijze

**Affiliations:** 1 Care and Public Health Research Institute (CAPHRI), Maastricht University Medical Center (MUMC), Maastricht University, Maastricht, Netherlands; 2 Research Centre for Midwifery Science, Zuyd University, Maastricht, Netherlands; 3 Department of Obstetrics and Gynaecology, University Medical Centre Rotterdam, Rotterdam, Netherlands

**Keywords:** midwife, qualitative research, maternity care, interventions, clinical decision-making

## Abstract

**INTRODUCTION:**

Appropriate use of interventions in maternity care is a worldwide issue. Midwifery-led models of care are associated with more efficient use of resources, fewer medical interventions, and improved outcomes. However, the use of interventions varies considerably between midwives. The aim of this study was to explore how knowledge and skills influence clinical decision-making of midwives on the appropriate use of childbirth interventions.

**METHODS:**

A qualitative study using in-depth interviews with 20 primary care midwives was performed in June 2019. Participants’ clinical experience varied in the use of interventions. The interviews combined a narrative approach with a semi-structured question route. Data were analyzed using deductive content analysis.

**RESULTS:**

‘Knowledge’, ‘Critical thinking skills’, and ‘Communication skills’ influenced midwives’ clinical decision-making towards childbirth interventions. Midwives obtained their knowledge through the formal education program and extended their knowledge by reflecting on experiences and evidence. Midwives with a low use of interventions seem to have a higher level of reflective skills, including reflection-in-action. These midwives used a more balanced communication style with instrumental and affective communication skills in interaction with women, and have more skills to engage in discussions during collaboration with other professionals, and thus personalizing their care.

**CONCLUSIONS:**

Midwives with a low use of interventions seemed to have the knowledge and skills of a reflective practitioner, leading to more personalized care compared to standardized care as defined in protocols. Learning through reflectivity, critical thinking skills, and instrumental and affective communication skills, need to be stimulated and trained to pursue appropriate, personalized use of interventions.

## INTRODUCTION

The appropriate use of interventions in maternity care has attracted considerable international attention^1,2^. It appears that medicalization of pregnancy and birth has negative consequences for women and babies, and results in higher health costs^2,3^. Therefore, medicalization of maternity care has become a contentious issue worldwide. There is a growing body of evidence that care provided by midwives results in fewer medical interventions, and increased satisfaction with the birthing experience without differences in adverse perinatal outcomes^4^. Regarding the appropriate use of interventions, using as little interventions as possible is not a purpose in itself, the purpose is to have the optimal balance between childbirth interventions and perinatal and maternal outcomes^3-5^.

Midwifery care provided by primary care midwives in the Netherlands attracts attention internationally, because of the high number of homebirths and the autonomy of midwives^6^. Two-thirds of Dutch midwives work as independent healthcare professionals in primary care, and are able to make, together with women, autonomous decisions about childbirth interventions or referral to obstetrician-led care^7^. Around 87% of the Dutch pregnant women start their prenatal care in primary midwifery care, however, during pregnancy and birth, a large percentage of women is referred to obstetrician-led care^8^. The referral percentage for nulliparous women is around 74% and 55% for multiparous women^8^.

For a long time, the leading principle of maternity care in the Netherlands has been that pregnancy and birth are physiological and normal processes^9^. Research exploring the background of midwives’ attitudes towards childbirth also suggests a common belief among Dutch midwives that pregnancy and childbirth are physiological processes and unnecessary use of interventions should be avoided^10,11^. Despite the fact that midwives seem to have a joint intention to promote physiological childbirth, different behaviors are seen towards clinical decision-making, resulting in variations in use of childbirth interventions including variations in referrals from midwife-led care to obstetrician-led care^12-14^.

Theories on human behavior, such as the Attitude, Social norms, Self-efficacy model (ASE-model), are relevant for studying intention and factors influencing behavior in human beings^15,16^. The ASE-model explains behavior by linking attitude, social norms and self-efficacy with behavioral intention and actual behavior. In addition to these three determinants of behavioral intention, factors such as ‘knowledge and skills’ and ‘barriers and facilitators’ also play a role in explaining behavior. Earlier studies in the Netherlands on determinants of intention and behavior towards clinical decision-making during childbirth, showed the influence of differences in midwives’ risk perception, work-experience, workload, setting (home or hospital), interaction with the woman, and regional protocols^10,12,13,17^. In addition, studies by Weltens et al.^13^ and Seijmonsbergen-Schermers et al.^14^ suggest that perspectives on birth as a physiological event differ between regions in the Netherlands.

In a previous study, we found that midwives with a ‘wait and see’ attitude seem to have a more restricted approach towards interventions compared to midwives with a ‘check and control’ attitude^11^. However, studies on the influence of knowledge and skills on midwives’ clinical decision-making are limited. Research on this subject is important because these are influenceable factors: knowledge and skills can be taught. This offers possibilities for behavioral change, contributing to appropriate use of interventions and improvement of the quality of midwifery care. Therefore, the objective of this study is to explore how knowledge and skills influence clinical decision-making of midwives towards the appropriate use of childbirth interventions.

## METHODS

### Study design

This study was part of a larger qualitative study exploring factors that may explain variations between midwives in decision-making on childbirth interventions^11^. We used in-depth interviews that combined a narrative approach with a semi-structured question route to indicate relevant topics (Table 1). In accordance with a narrative approach, we invited the participating midwives to elaborate and share their stories about situations during pregnancy, birth, and postpartum period, where the use of interventions was an issue. The narrative approach made it possible to explore, from a broader perspective, how midwives used their knowledge and skills during clinical decision-making^18^.

**Table 1:** Question route for interviews

1. What do you think is the definition of a medical intervention in midwifery care?
2. Please describe a situation during maternity care (pregnancy, birth or postpartum) in which many interventions were performed.
3. Please describe a situation during maternity care (pregnancy, birth or postpartum) in which few interventions were performed.
4. Which factors influence whether or not a medical intervention is performed?

### Setting

In the Netherlands, low-risk pregnant women can choose to give birth at home, in a birth center, or in hospital under the supervision of their independent midwife. Women will not receive interventions such as epidural analgesia, augmentation, or continuous fetal monitoring, while in primary midwife-led care. If a woman wants these interventions or if they become necessary, a referral to obstetrician-led care is indicated. Therefore, referral to obstetrician-led care is seen as an intervention in this study. Criteria for referral are described in the List of Obstetric Indications^9^.

### Participants

Participants were purposive sampled and included midwives from midwifery practices with either a low or a high use of childbirth interventions in order to explore differences between these two groups. The definition of low or high use of childbirth interventions is based on literature describing variations in childbirth interventions^14,19^. For the purpose of this study we used data from the Dutch national perinatal register (Perined), to identify midwifery practices with a high or a low intervention rate (https://www.perined.nl). Practices in the group with a low intervention rate had a combination of three factors: low referral rate (<35th percentile), a high homebirth rate (>65th percentile), and a low episiotomy rate (<35th percentile). Practices considered as having a high use of interventions had the combination of: a high referral rate (>65th percentile), a low homebirth rate (<35th percentile) and a high episiotomy rate (>65th percentile).

We invited midwives from 46 midwifery practices, 23 for each category (low and high use of interventions), taking into account geographical locations and practice sizes to include various types of practices throughout the Netherlands. We intended to interview one midwife per practice. We send a reminder two weeks after the invitation.

### Data collection

Before the interviews, we distributed a short questionnaire to collect the participants’ demographic characteristics. The face-to-face interviews were conducted in June 2019 by four interviewers with a midwifery background, and without a personal relationship with the participants. It was unknown to the interviewers to which of the two categories the participant belonged. The first two interviews of each interviewer were observed by another member of the research team to ensure validity, consistency and enhance quality across each of the interviews. After the first four interviews, the research team made small adjustments to the semi-structured question route, in order to reinforce the narrative approach. The interviews were audio-recorded and transcribed verbatim for analysis. Each participant received a transcription of the interview for a member-check. We anonymized and encrypted the transcripts, together with field notes, the data were safely stored and only accessible for the research team.

### Ethical considerations

According to the ‘Act governing research involving human subjects’ in The Netherlands (WMO), formal ethical approval by a research ethics committee is only required for medical research where participants are subject to interventions or procedures, or are required to follow specific, research-related rules of behavior^20^. None of these applies to this research. A self-assessment tool from the Medical Ethics committee of Maastricht University, The Netherlands, signaled our study as exempted from formal medical ethical review^21^. Written consent was obtained from all participants before participating.

### Data analysis

We analyzed the data using deductive content analysis^22^. The goal of this analysis process was to gain deeper knowledge about the aspects involved in clinical decision-making for the use of interventions in the two groups with a different use of interventions. We used the ASE-model^16^ as a theoretical framework, focusing on determinants of knowledge and skills. Throughout data analysis, we found that in the communication skills, a distinction could be made between instrumental and affective communication, as identified in theories on healthcare communication^23,24^. Instrumental communication is task-related behavior and involves skills such as asking questions and providing information, while affective communication is socio-emotional behavior and involves skills such as reflecting feelings and showing empathy and concern^24^. We extended the theoretical framework using these findings complementary to the ASE-model.

The first author read and reread the complete transcripts of each interview to identify any descriptions related to the framework. The analysis process was open to identify any new themes that would emerge from the data. The second author conducted a dependability and conformability audit to check the analysis against accepted standards and examine the analysis process and records for accuracy. After nine interviews, we reached saturation on the level of themes and subthemes. We analyzed the remaining nine interviews to check whether any codes or themes had been missed and any falsifying findings could be found, also confirming the stated themes. We used the online software program Dedoose version 8.3.17 and recorded the study’s procedure in a logbook. The standards for reporting qualitative research (SRQR) gave guidance (Supplementary file) to the writing of the current article^25^.

## RESULTS

In total, 22 midwives accepted the invitation for an interview. Two midwives were excluded; one midwife because a colleague from the same midwifery practice was already interviewed, and a second midwife because no suitable date or time could be planned. Thirteen midwives worked in a midwifery practice with a low use of interventions and seven in a midwifery practice with a high use of childbirth interventions. The reasons for the non-response of the 24 remaining practices are unknown. Participants varied in terms of years of midwifery experience, place of education, and practice characteristics including geographical location (Table 2).

**Table 2:** Characteristics of the participating primary care midwives with low and high use of childbirth interventions, working in the Netherlands (N=20)

*Characteristics*	*Low use (n=13)*	*High use (n=7)*
**Working experience** (years)		
<10	2	2
10–20	5	2
20–30	3	3
>30	3	0
**Place of midwifery training**		
The Netherlands	12	4
Abroad (Belgium, UK, Switzerland)	1	3
Practice size*		
<80	6	0
80–300	5	4
>300	2	3
**Size of the midwifery team in the practice**		
1–2	6	1
3–4	4	3
≥5	3	3

* Size of the midwifery practice in number of women receiving complete care (pregnancy, birth and postpartum) annually.

We identified three main themes: 1) Knowledge – learning through reflectivity, 2) Critical thinking skills – advanced knowledge in context, and 3) Communication skills – making your knowledge work. An overview of the main themes and subthemes is given in Table 3.

**Table 3:** Overview of main themes and subthemes

**1. Knowledge – learning from different sources**
a. Getting the basics
b. Deepening the knowledge base
Learning through experience
Reflection on evidence
**2. Critical thinking skills – advanced knowledge in context**
**3. Communication skills – making knowledge work**
a. Communication with women: giving and gaining background information
Instrumental communication
Affective communication
b. Communication with colleagues: the ability to speak up

### Knowledge - learning from different sources

The midwives in our study described how their knowledge on the use of interventions developed from the basics towards a deeper understanding.

*Getting the basics*

Midwives from both high and low intervention groups elaborated how their use of interventions is first of all based on knowledge acquired during their midwifery training. Parameters for a physiological progress of pregnancy and birth were taught, which they used for the rest of their working life:

*‘They [educators] really taught me to act according to the physiology. They also taught me to show what you can do as a primary care midwife.’* (Midwife 7)

In the Netherlands, half of the 4-year midwifery education program consists of clinical placements in both primary care midwifery practices and hospital settings. Midwives from both groups reported that their clinical decision-making and use of interventions is strongly influenced by these placements, especially the final placement before graduation. Some midwives described how they replicate the clinical decision-making style of the midwifery practice where they had taken their final placement. Either because they believed this was the appropriate care to provide, or as habitual standard. Some participants mentioned the lack of specific experiences during their placements. For example, midwives questioned if knowledge about a topic such as the pros and cons of different birth places and how you discuss this information with the woman and her partner, is sufficiently embedded in their study program:

*‘If you don't learn during your placement to discuss in an open way, and also mention the benefits of giving birth at home [….], you will hear that once during the study program, I hope, but it will not become normal in your way of working and giving information.’* (Midwife 10)

*Deepening the knowledge base*

Midwives in our study described how, over time, experience and reflection deepened their knowledge.

*Learning through experience*

A second source of knowledge was knowledge acquired through practical experience by working as a midwife. Midwives in the study spoke about how their experiences with a cascade of interventions after administration of epidural analgesia, a fetal growth scan, or the diagnosis of gestational diabetes, deepens their understanding of the consequences of using interventions:

*‘Especially by epidural analgesia. An IV is placed, followed by a catheter, an electrode on the head of the baby, laying down in the bed; the hospital's “total package”. It all causes a lot of misery…’* (Midwife 2)

In particular, midwives from the group that utilize low rates of childbirth interventions as part of their clinical practice spoke about the added value of knowledge from experience. Because of this knowledge, they gained confidence in the natural process of birth, and became more restrained in using interventions. Most midwives from the group that utilize low rates of childbirth interventions as part of their clinical practice reported that their experiences create the possibility to tailor the use of interventions to the individual situation, instead of following standardized recommendations suggested by national guidelines or local protocols:

*‘The longer you work, the easier it becomes to feel free in decision-making, and to decide what is best in each situation.’* (Midwife 12)

*Reflection on evidence*

Some midwives working in practices with a low use of childbirth interventions described situations in which they critically reflect on their own clinical decision-making. These midwives mentioned scientific literature they read and interpret to make clinical decisions regarding childbirth interventions in situations where no guideline or consensus exists:

*‘In our practice we say “the less you do, the more beautiful the birth will be”. It's a kind of conscious choice […] It's also very clearly written in the Lancet series. A lot of midwives use amniotomy to accelerate labor, and then you think: “why would you want to do that?” […] Eventually, it becomes evident that it does not help at all.’* (Midwife 1)

In addition, midwives in the group that utilize low rates of interventions as part of their clinical practice described how they question assumptions and generate knowledge through reflection:

*‘Ultrasounds are absolutely interventions. Enormous interventions, based on which, care pathways can go in all directions. You should be very careful with the use of them. Not a standard thirty-week fetal growth scan […]. I also don't understand that colleagues go along with this. If you think about it carefully and read all the evidence about it.’* (Midwife 10)

In contrast, none of the midwives in the group that utilize high use of childbirth interventions as part of their clinical practice gave examples of using scientific literature in their clinical decision-making process. They reported how they often align with local protocols and do not use national guidelines. They accommodated to the local agreement to use these protocols, which were different from the national guidelines, often leading to a more interventionist approach.

Overall, midwives described that the formal midwifery education program is an important source of knowledge. In addition, midwives with a low use of interventions clearly described how they have extended their knowledge, and the application of knowledge by reflecting on experiences and evidence. This extended knowledge through reflection influenced their clinical decision-making. In contrast, midwives with a high use of interventions did not mention this reflection, but described how they adhere to local protocols for their decision-making.

### Critical thinking skills – advanced knowledge in context

All midwives felt competent to perform interventions, such as vaginal examination or amniotomy. A difference seemed to exist in the process leading up to the use of these interventions; a difference in the reflective process involving critical thinking skills to make well-founded decisions during midwifery care. Midwives who utilize low rates of interventions as part of their clinical practice spoke about how they continuously reflect on the ongoing situation and constantly ask themselves if it is necessary to intervene. Such reflective moments seem to contribute to less standardized application of interventions compared to the group of midwives with a high use of childbirth interventions who did not mention reflective moments:

*‘During childbirth I try to say to myself: “hold back, you don't have to [artificial rupture the membranes]. The woman does not benefit from that. She will only have more severe contractions. That's of no benefit”.’* (Midwife 20)

Midwives who utilize high rates of interventions as part of their clinical practice described how they apply interventions to control the process of labor or because this is the standard procedure:

*‘We make a fetal growth scan at 30 weeks. That's more because it makes us feel safe. And because we know we detect more small babies. We just want to check it.’* (Midwife 17)

Summarizing, midwives in the group that utilize low rates of interventions as part of their clinical practice seemed to use critical thinking skills for a reflective process where appropriate use of interventions is being pursued. They have a reflection moment in action, which makes them wait and evaluate whether an intervention is beneficial at that moment. Such moments were not described by midwives in the group that utilize high rates of interventions.

### Communication skills – making knowledge work

In the interviews, midwives stated how the communication skills they use in their interaction with women are differed from those they use in their interaction with colleague healthcare professionals.

*Communication with women: giving and gaining background information*

In their communication with women, the midwives in our study use instrumental and affective communication.

*Instrumental communication*

All midwives in our study used instrumental communication to explain information to women. However, midwives who utilize low rates of interventions as part of their clinical practice explained that they communicate extensively about treatment options to facilitate women to make an informed choice. Some of them spoke about open communication, including conversations about uncertainties or their own professional experiences with specific interventions, such as induction of labor, ultrasound, and fetal monitoring. These midwives also expressed a broader view on childbirth interventions and the role of healthcare professionals in the application of interventions:

*‘In general, we find it easier to give more [interventions] than to give less [interventions]. Healthcare providers see more danger in not intervening than in intervening. I think it is my job to make this clear to the woman and her partner.’* (Midwife 12)

Midwives who utilize high rates of interventions as part of their clinical practice seem to employ a different communication style regarding treatment options. They discussed about how they suggest a certain care pathway and ask the woman if she agrees with this plan. These midwives seem to provide only limited information about a selection of care options that fit in the care pathway, suggesting that they use an opt-out approach when outlining the treatment plan for pregnancy and birth:

*‘In case of a high maternal body mass index or someone who has used drugs, I assimilate the individual care pathway according to these risk factors. I always go through everything with the client and then say: “let's do fetal growth ultrasounds anyway, and an OGTT”. That's what I explain.’* (Midwife 17)

*Affective communication*

Midwives who utilize low rates of interventions as part of their clinical practice indicated also a range of affective communication skills they use when interacting with pregnant women. These midwives spoke about situations they had encountered where women had requested more or fewer interventions than suggested by the national guidelines or local protocols. They described how they took time to actively listen to these women and started a conversation to investigate the underlying motives for the request:

*‘Someone may say that she wants to give birth at home, even when it's preterm. But then I still know nothing. Is she traumatized? Would she like to experience this once? […] Actually, the conversation starts at that point. You need to know a lot more.’* (Midwife 12)

In contrast, midwives who utilize high rates of interventions as part of their clinical practice seemed less inclined to investigate underlying motives in such situations, and described how they quickly shift to arranging practical matters or to refer to obstetrician-led care. They discussed situations where women requested fewer interventions then recommended in guidelines or local protocols, and how they usually do not grant such requests. If a woman requested more interventions, for example an induction of labor, they arranged a consultation in obstetrician-led care without exploring the woman’s motives extensively:

*‘She was pregnant of her third child, an unplanned pregnancy, and she was anxious from the beginning. She asked for a planned cesarean. So, we have sent her to the obstetrician at an early stage of pregnancy.’* (Midwife 8)

*Communication with colleagues: the ability to speak up*

Midwives who utilize low rates of interventions as part of their clinical practice seem to use more persuasive communication strategies during interaction with other healthcare professionals, such as other midwives, residents and obstetricians. They described how, in discussions, they are comfortable to deal with resistance or disagreement from other healthcare professionals and are prepared to defend their point of view. These midwives saw it as their duty to be the advocate for the woman’s wishes. They reported how it is necessary to be persistent in these discussions to achieve physiological care or the care the pregnant woman desires:

*‘During multidisciplinary meetings we are transparent and open. We honestly say what we think and what we want. […] Always as the advocate of the woman. It is very important that you neatly organize the desired healthcare plan for her.’* (Midwife 1)

Some midwives who utilize high rates of interventions as part of their clinical practice described how they avoid discussion with healthcare professionals in the hospital, especially with obstetricians. Generally, they conformed to the wishes of the local obstetricians:

*‘If someone has an Hb [hemoglobin] of 5.9, then you don't have to do anything according to the national guidelines. But the obstetrician is not very happy when he gets someone on his operation table with an Hb of 5.9. […] So if it gets towards 6.0, we prescribe iron. Because we know the obstetrician doesn't like it, you know.’* (Midwife 4)

Overall, the results suggest that midwives who utilize low rates of interventions as part of their clinical practice explore women’s options and considerations to a higher extent, using a communication style that balances instrumental and affective communication skills, compared to midwives who utilize high rates of interventions as part of their clinical practice. In addition, the narratives of the midwives suggest that midwives who utilize high rates of interventions are less skilled to engage in discussions with colleague healthcare professionals.

## DISCUSSION

In this qualitative study, we explored how knowledge and skills influence clinical decision-making towards the appropriate use of childbirth interventions. We found that the level of reflection seems to differ among midwives with either a low or high use of childbirth interventions. Midwives who utilize low rates of interventions as part of their clinical practice described how they reflect on previous experiences and the evidence that influence their clinical decision-making. In addition, they seem to use more critical thinking skills during reflective moments as well as a communication style that balances instrumental and affective communication skills in interaction with the woman resulting in more personalized care. This personalized approach to maternity care may help in the pursuit for an appropriate use instead of routine use of interventions and may reduce medicalization in childbirth.

### Reflective practitioner

Our study suggests that the knowledge and skills of midwives who utilize low rates of interventions as part of their clinical practice resemble those of a reflective practitioner. A reflective practitioner is someone who ‘lives’ reflection as a way of ‘being’ rather than just ‘doing’^26^. Reflective practice is linked to the concept of learning through and from experiences, by actively analyzing and questioning choices and decisions. Individual healthcare practitioners who are aware of what they are doing and critically evaluate their own responses to situations are reflective practitioners. This reflectivity helps them to provide appropriate interventions, to the right person at the right time^26,27^. This also emerged in our study, where midwives with a higher tendency to reflect on provided care were less inclined to provide standardized care.

Reflection on practice is an important skill for a reflective practitioner, however, reflection in practice is also important. Lake and McInnes^28^ describe that critical thinking skills help midwives in their clinical judgment and clinical decision-making, and enable them to provide appropriate, woman-centered and evidence-based care^28^. In our study, midwives who utilize low rates of interventions as part of their clinical practice described reflective moments in care, where they consciously consider different options for clinical decision-making. During this reflection-in-action, they used critical thinking skills to make a balanced decision whether the intervention is beneficial at that moment. Previous studies have shown that higher interventions rates do not automatically lead to better perinatal outcomes^29^. The reflective approach towards interventions can help in the pursuit for appropriate use of interventions.

Important elements of reflection on practice are the recognition of non-evidence based care, and to search and interpret evidence for clinical decision-making^30^. These skills are crucial to practice physiological care with an appropriate use of interventions^30,31^. In our study, midwives who utilize high rates of interventions as part of their clinical practice did not describe using scientific literature in their clinical decision-making process. It is possible that they lack skills to assess evidence and to recognize non-evidence based care.

Other elements of reflective practice are: the skill to discuss and debate within the multidisciplinary setting of maternity care^30,31^; and the ability to speak up and to persuasively communicate the wishes of women and the advantages of a physiological birth. Midwives should be able to effectively communicate considerations in clinical decision-making, including available evidence with other healthcare professionals^6^. These skills seem less present in the group with a more interventionist approach. When a midwife is less skilled to speak up and advocate the midwifery philosophy of care, the risk philosophy of obstetrician-led care will be predominant, making it more likely that a higher use of interventions will occur^32^.

Reflection has been described as an important learning strategy for professionals to create awareness of their own skills and attitude on the actual performance^33^. Probably, explication of reflective skills and training of these skills can enhance the reflectivity of midwives^34^. The Optimality Index – Netherlands (OI-NL) is a tool that can support reflection on maternity care practices from a physiological perspective and facilitate optimal birth practices: maximal outcome with minimal intervention^30^.

### Effective communication: The balance between communications skills

Midwives who utilize high rates of interventions as part of their clinical practice seem mainly focused on the need to provide information by using instrumental communication, which fits with an informed consent approach. Midwives who utilize low rates of interventions as part of their clinical practice showed additional attention for women’s need to feel known, by using more effective communication skills and gaining insight into women’s knowledge and motives. Such a balance between both communication styles is needed to invest in an effective partnership between woman and midwife^24,25^, and is more in line with the model of shared decision-making (SDM)^35^. However, an informed consent approach can unjustly be mistaken for SDM by care professionals, because they ask for assent but there is no dialogue as medium for the decision-making process^35^. In our study, we observed that midwives, mainly in the group with a high use of interventions, used informed consent instead of offering relevant knowledge on various options and working together with the woman to establish choices that fit her circumstances. Applying SDM means that a midwife explains the various options and their evidence base. This makes clinical decision-making less dependent on personal beliefs of the individual midwife^35^, and leads to more awareness about appropriate use of interventions instead of standardized use. Thomas et al.^36^ emphasizes that major changes are necessary in educational structures and maternity care systems to promote critical reflexivity required for SDM^36^. This supports our findings that these skills need further development for care providers to be fully competent.

### Strengths and limitations

We used purposive sampling and included a diverse population of midwives in terms of years of midwifery experience, place of education, and midwifery practice characteristics. In total, seven midwives who utilize high rates of interventions as part of their clinical practice participated in this study and thirteen midwives who utilize low rates of interventions as part of their clinical practice participated. This unequal distribution of participants possibly might have influenced the results of this study, because midwives in the low intervention group already work as a reflective practitioner. Factors such as team size, and place of midwifery training might influence clinical decision-making, but was not investigated in this study. We reached data saturation in both groups. Attention was paid to the methodological rigor, with a reflective journal being kept by the first author and all key decisions during data collection and analysis being peer reviewed by the second author. Complementary, we re-read the interviews after we finalized the findings, to verify the results and limit bias.

A limitation of this study was that midwives could only be included based on the practice level of interventions, because the Perined database cannot be analyzed on the level of individual midwives. Therefore, the assumption was made that individual midwives provide care in accordance with the level of childbirth interventions of the midwifery practice they work at. However, midwives are autonomous healthcare professionals and make individual decisions whether to perform an intervention or not. We cannot rule out that some misclassification took place. However, the interviewers were blinded for this classification, and we observed no signs of misclassification during the analysis of both groups.

## CONCLUSIONS

The results of this study suggest that there are differences in knowledge and skills between primary care midwives, probably influencing clinical decision-making and the use of childbirth interventions. The knowledge and skills of a reflective practitioner seem to lead to more personalized care compared to standardized use of interventions as defined in protocols. This personalized care helps in the pursuit for appropriate use of childbirth interventions and may reduce medicalization in childbirth. Reflection on experiences and evidence, a balanced communication style with instrumental and effective communication skills, and the use of critical thinking skills during reflection-in-action, need to be taught and trained to midwives to pursue an appropriate and personalized use of interventions.

## Data Availability

The data supporting this research are available from the authors on reasonable request.
